# Radiation-induced angiosarcoma: case report

**DOI:** 10.31744/einstein_journal/2020RC5439

**Published:** 2020-11-25

**Authors:** Lucas Aguiar Alencar de Oliveira, Antonio Fortes de Pádua, Maria Adélia Medeiros e Melo, Elisa Rosa de Carvalho Gonçalves Nunes Galvão, Mharcus Carneiro Vieira, Jerúsia Oliveira Ibiapina, Danilo Rafael da Silva Fontinele, Sabas Carlos Vieira

**Affiliations:** 1 Universidade Federal do Piauí TeresinaPI Brazil Universidade Federal do Piauí, Teresina, PI, Brazil.; 2 Hospital São Marcos TeresinaPI Brazil Hospital São Marcos, Teresina, PI, Brazil.; 3 Universidade Estadual do Piauí TeresinaPI Brazil Universidade Estadual do Piauí, Teresina, PI, Brazil.

**Keywords:** Hemangiosarcoma, Breast neoplasms/radiotherapy, Mastectomy

## Abstract

Angiosarcoma of the breast accounts for less than 1% of breast tumors. This tumor may be primary or secondary to previous radiation therapy and it is also named “radiogenic angiosarcoma of the breast”, which is still a rare entity with a poor prognosis. So far, there are only 307 cases reported about these tumors in the literature. We present a case of a 73-year-old woman with a prior history of breast-conserving treatment of right breast cancer, exhibiting mild pinkish skin changes in the ipsilateral breast. Her mammography was consistent with benign alterations (BI-RADS 2). On incisional biopsy specimens, hematoxylin-eosin showed atypical vascular lesion and suggested immunohistochemisty for diagnostic elucidation. Resection of the lesions was performed and histology showed radiogenic angiosarcoma. The patient underwent simple mastectomy. Immunohistochemistry was positive for antigens related to CD31 and CD34, and C-MYC oncogene amplification, confirming the diagnosis of angiosarcoma induced by breast irradiation. A delayed diagnosis is an important concern. Initial skin changes in radiogenic angiosarcoma are subtle, therefore, these alterations may be confused with other benign skin conditions such as telangiectasia. We highlight this case clinical aspects with the intention of alerting to the possibility of angiosarcoma of the breast in patients with a previous history of adjuvant radiation therapy for breast cancer treatment. Sixteen months after the surgery the patient remains asymptomatic.

## INTRODUCTION

Angiosarcoma of the breast accounts for less than 1% of breast tumors. It may be primary or secondary to previous radiation therapy and/or chronic lymphedema after breast cancer treatment.^(^^1^^)^

Secondary angiosarcoma of the breast is generally related to breast radiation therapy and is termed radiogenic angiosarcoma of the breast (RASB). The absolute risk of developing RASB is low, less than 0.5% and the relative risk of developing the condition is 15.9 in patients who received breast radiation therapy.^(^^2^^)^

Early alterations in RASB are subtle and may be confused with other benign skin conditions such as telangiectasia, which may not alert the physician to the diagnosis.

We present a case of RASB and discuss diagnostic and treatment aspects.

## CASE REPORT

**Figure 1:**
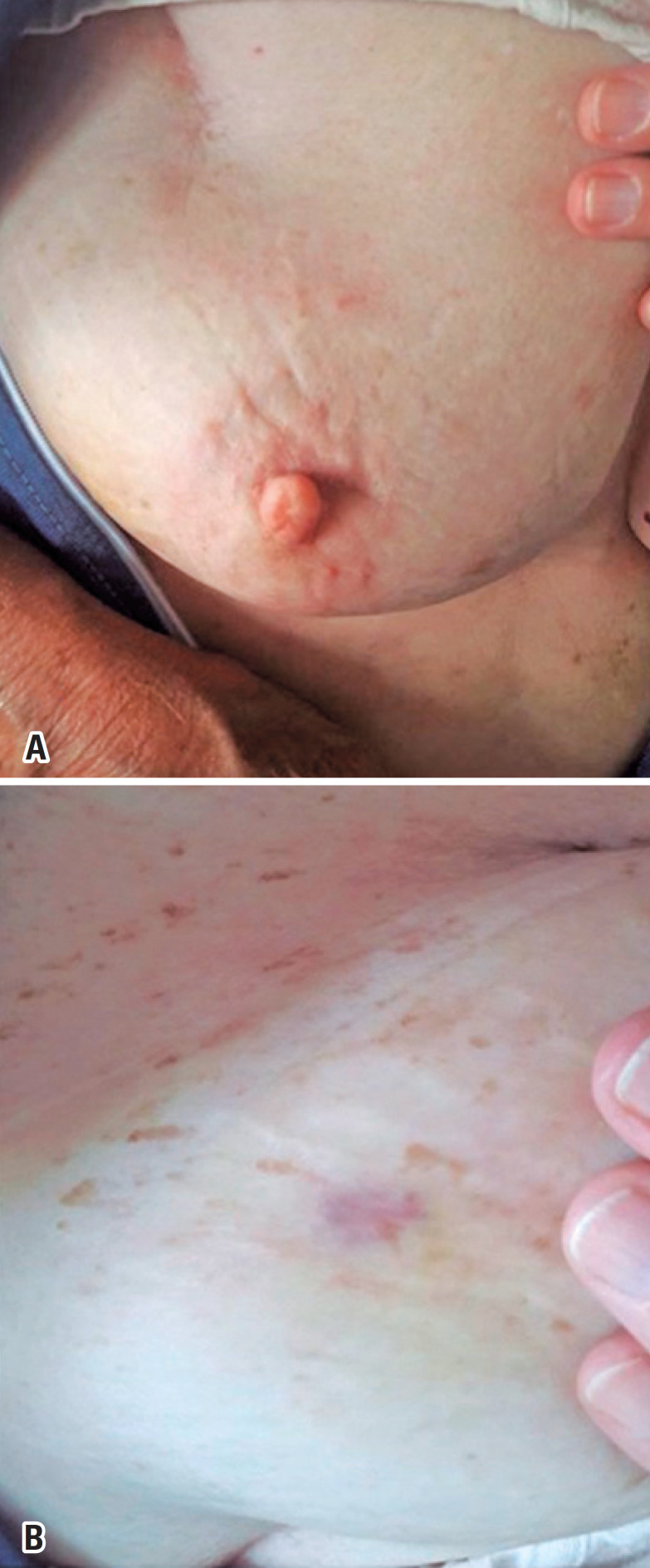
Pinkish/violaceous lesions being one located at the junction of the upper quadrants (A) and another at the junction of the lower quadrants measuring 0.5cm (B)

A Caucasian, 73-year-old woman sought the breast disorder division with the complaint of a pinkish skin change in her right breast for about 30 days. A bilateral mammogram had been performed five months previously, showing benign alterations ( *Breast Imaging Reporting and Data System* – BI-RADS™ 2). The patient had a personal history of a G3 invasive right breast carcinoma of no special type, treated with segmental resection and axillary lymph node dissection level I and II on December 2011. At the time of the operation, the tumor measured 3.0cm and three axillary lymph nodes had metastases (3/10). Immunohistochemistry showed that the tumor was estrogen receptor positive (90%), progesterone receptor positive (2%), HER-2 negative and Ki-67 positive (60%), luminal B subtype. Adjuvant therapy of the patient included six cycles of doxorubicin, cyclophosphamide and paclitaxel chemotherapy followed by radiation therapy (25 sessions with a 50Gy dose to the whole right breast and supraclavicular fossa, in addition to a 10Gy boost). She is currently undergoing endocrine therapy with letrozole (sixth year of treatment). On physical examination, the patient had two pinkish-violaceous lesions in the right breast, one that was mildly erythematous and almost imperceptible at the junction of the upper quadrants (UQJ) and another more intensely violet lesion at the junction of the lower quadrants (LQJ) measuring 0.5cm ( Figure 1 ).

**Figure 2:**
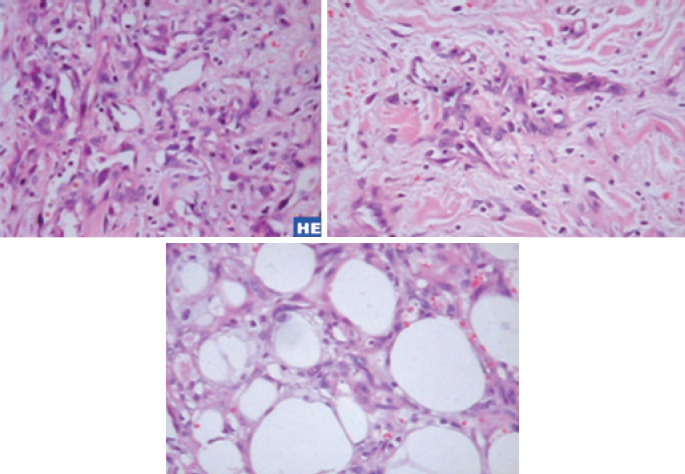
Pathological anatomy

**Figure 3:**
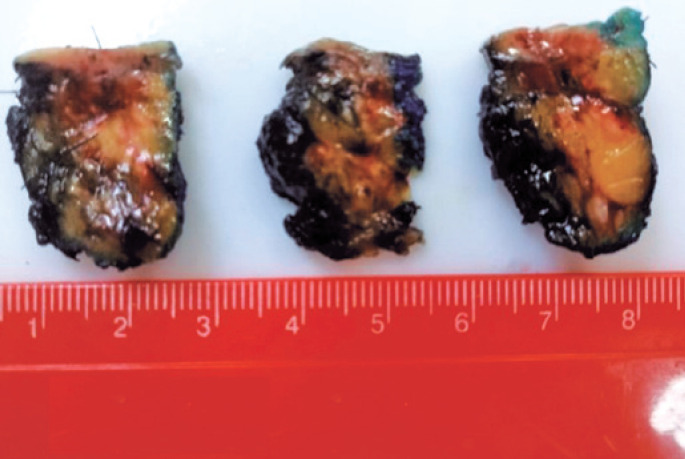
Specimens obtained from skin lesions resected at the junction of the upper quadrants and junction of the lower quadrants of the right breast

**Figure 4:**
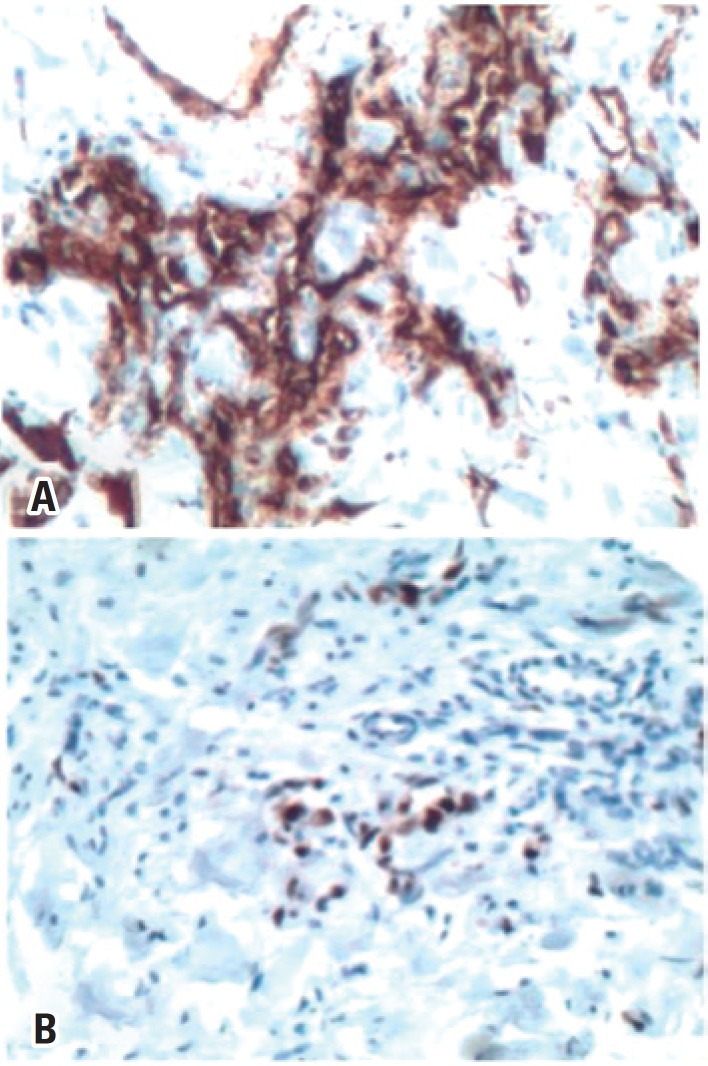
Immunohistochemical study showing positivity for (A) cluster of differentiation 31, and (B) oncogene C-MYC

An incisional biopsy of the lesion in the LQJ was performed and demonstrated an atypical vascular lesion on hematoxylin-eosin stain. A complementary immunohistochemical study was suggested for diagnostic conclusion. Due to this result, the patient underwent a wide resection of both cutaneous lesions. In the meantime, immunohistochemical study result revealed an angiosarcoma. Anatomic pathological report of the resected specimens showed a well-differentiated angiosarcoma (G1), a neoplasm characterized by anastomosing vascular ducts lined by atypical endothelial cells characterized by hyperchromatism and anisocariosis, sometimes containing red blood cells, arranged in an infiltrative growth pattern, permeating the mammary parenchyma and dermis ( Figure 2 ) at the UQJ measuring 1.9x1.4cm, and at the LQJ a neoplasm restricted to the dermis, measuring 1.1x0.5cm ( Figure 3 ).Immunohistochemical revealed positivity for the expression of cluster of differentiation 31 (CD31) and oncogene C-MYC (C-Myc), confirming malignancy secondary to radiation therapy ( Figure 4 ). Computed tomography of the chest and abdomen and bone scintigraphy showed no signs of distant metastases. The patient underwent a right simple mastectomy without reconstruction as complementary treatment. The final histopathology report showed a well-differentiated angiosarcoma (G1) and three more microscopic foci were found in the breast parenchyma, the largest measuring 2mm. Two years after surgery, the patient has no evidence of disease recurrence.

## DISCUSSION

As opposed to primary angiosarcoma of the breast that affects young women, mean patient age at diagnosis of RASB was 70 years and the mean latency period between radiation therapy and diagnosis was 6 years.^(^^3^^)^ The pathogenesis of RASB is still unclear.^(^^4^^)^

The two largest published case series of RASB, one with 79 cases and another with 95 cases reported that the 5-year disease-free survival (DFS) was 47% and 62.6%, respectively.^(^^5^^,^^6^^)^

However, RASB has a high local recurrence rate and metastasis is more common to the lungs and liver, occurring simultaneously or soon after local recurrences.^(^^7^^)^

Clinical manifestations of RASB are frequently described as small, violaceous skin lesions, which resemble telangiectasia. Nevertheless, nodules, plaques or patches may also appear.^(^^3^^)^

Imaging of angiosarcoma is nonspecific. Mammography and ultrasonography have no pathognomonic features. Magnetic resonance imaging is considered the most promising imaging test for malignancy patterns.^(^^8^^)^

Diagnosis of angiosarcoma is made by biopsy. Histological features of primary angiosarcoma of the breast RASB are indistinguishable, except for the more common cutaneous involvement in RASB, as well a higher proportion of less-differentiated epithelioid tumors.^(^^8^^,^^9^^)^ To date, three main histopathologic grades have been described for angiosarcoma: low-grade or type 1 (G1), intermediate-grade or type 2 (G2) and high-grade or type 3 (G3).^(^^1^^)^ D'Angelo et al.,^(^^6^^)^ have shown that tumor grade does not seem to have prognostic value and even low-grade lesions may metastasize. Espat et al.,^(^^10^^)^ consider that all angiosarcomas associated with radiation are considered high-grade tumors. Thus, the tumor grade has no prognostic value in breast angiosarcomas.

On immunohistochemistry, angiosarcomas are positive for antigens related to CD31, CD34 and sometimes podoplanin for the diagnosis of less-differentiated tumors.^(^^6^^)^ Laé et al.,^(^^11^^)^ found a 5-to 20-fold amplification of the C-MYC in all angiosarcomas induced by breast irradiation. These data may provide a basis for additional targeted therapy.^(^^11^^)^ The expression and amplification of c-MYC in RASB is also important in the differential diagnosis of benign lesions named atypical vascular lesions. Positivity of antigens related to CD31 and CD34 confirms the diagnosis of angiosarcoma and expression and amplification of cC-MYC are shown in RASB.^(^^3^^)^

From the molecular point of view, it is assumed that point mutations in BRCA2 are causes of some secondary angiosarcomas of the breast. The loss of function of BRCA mutated prevents to exert protection against radiation-induced DNA damage.^(^^12^^)^ West et al.,^(^^13^^)^ presented a case report in which a patient with BRCA2 who developed chest wall angiosarcoma after mastectomy. Later, Kadouri et al.,^(^^14^^)^ reported the genetic evaluation of three cases of secondary breast angiosarcoma, two BRCA1 and one with BRCA2 and one without. They estimated an approximately twice as high risk of angiosarcoma in patients with BRCA1/2. However, this risk should not be considered in the irradiation treatment of this mutated population.^(^^12^^)^

There is no gold standard for surgical treatment of angiosarcoma. A wide local resection or mastectomy is the most commonly performed treatment. Simple mastectomy is the surgery of choice. It is debatable whether axillary dissection is required, since nodal involvement is uncommon.^(^^5^^)^

In general, chemotherapy regimen is chosen empirically due to the rarity of the disease and lack of definite standardized treatment. Some studies^(^^5^^,^^9^^)^ have suggested that treatment with anthracyclin-based chemotherapy, with either doxorubicin, or epirubicin with ifosfamide may improve both disease-free DFS and overall survival (OS). Systemic chemotherapy and re-irradiation are indicated only in RASB patients with local and/or systemic recurrences.^(^^3^^)^

Table 1 present case report studies and case series (up to 10 cases) on radiation-induced breast angiosarcoma after treatment for breast cancer from the last five years (2015-2020) indexed in PubMed.gov .

**Table 1:** Radiation-induced breast angiosarcoma after treatment for breast cancer^(^^15^^-^^39^^)^

Author/year	Sex/ age (years)	Number of cases	Primary tumor treatment	Size of angiosarcoma/previous radiotherapy time	Follow-up (months)	Result	Angiosarcoma's treatment	Recurrence/treatment
Abbenante et al.^(^^15^^)^	F/ 70	1	BS + L + RT	14 years	4	No disease	S	
Shiraki et al.^(^^16^^)^	F/72, F/80	2	S + RT/S	18cm/5 years, 3 years	32/ 17	Deaths/partial response	S/S	Yes/CT
Jayarajah et al.^(^^17^^)^	F/62	1	BS + L + RT	0.5cm/5 years	15	No disease	BS + CT	
Lewcun et al.^(^^18^^)^	F/64	1	BS + CT + RT	0.8cm 6 years	24	Complete response	NA QT + BS	
Kong et al.^(^^19^^)^	F/75	1	S + L + CT+ RT + H	5.6cm/20 years	15	No disease	S + RT	
Suzuki Y et al.^(^^20^^)^	F/62	1	S + RT+ H	8 years	8	No recurrence	S + CT	Yes
Amajoud et al.^(^^21^^)^	F/73 *	10	S + RT	10cm/7.3 years *	13 *	5 deaths and 5 with no disease	S + CT + RT	Yes
Lee et al.^(^^22^^)^	F/72	1	S + RT	6 years				
Verdura et al.^(^^23^^)^	F/79	1	S + L + CT + RT	2cm 8 years	12	No disease	NA QT + S	
Tsapralis et al.^(^^24^^)^	M/72	1	S + L + RT	6 years		Death	S + ECT + CT	Yes
Wei et al.^(^^25^^)^	F/39	1	S + RT	4 years			S	
García Novoa et al.^(^^26^^)^	F/37	1	BS + L + CT + H + RT	0.5cm/4 years			S	
Bonzano et al.^(^^27^^)^	F/57	1	S + L + RT	10cm/8 years	30	No disease	S + CT + RT	
Farran et al.^(^^28^^)^	F/67	1	S + L + RT	1cm/8 years		No disease, still in follow-up	S	
Disharoon et al.^(^^29^^)^	F/ 68	1	S + L + RT	1cm/9 years			S	
Plichta et al.^(^^30^^)^	F/72	1	S + RT	10cm/5 years	12	No disease	S + CT	
Tato-Varela et al.^(^^31^^)^	F/62	1	S + L + RT	1cm/8 years	0.5	Asymptomatic	S	
Wronski et al.^(^^32^^)^	F/56	1	S + L + RT	5 years	0.06	Asymptomatic	S	
Wilhelm et al.^(^^33^^)^	F/70 *	7	S + RT	8.5 years *				
Mocerino et al.^(^^34^^)^	F/77	1	S + H + RT	2cm		No disease	S + ECT + RT + CT	Yes/RT + CT
Peterson et al.^(^^35^^)^	F/72	1	S + RT	1.5cm/14 years	20	No disease	S	
Tidwell et al.^(^^36^^)^	F/68	1	RT	3cm/9 years			S	Yes
Uryvaev et al.^(^^37^^)^	F/78 *	6	S + RT	9.2 years	41.8	4 with no disease, 1 Death, 1 at CT	S + CT + 3 RT	Yes (3)
Parvez et al.^(^^38^^)^	F/55	1	S + L + CT + RT + H	1.5cm/0.5 years			BS + RT	
Styring et al.^(^^39^^)^	F/54.5 *	6	S + RT + 2 CT	7 years *			S	

* Average

F: female; BS: bilateral surgery; L: sentinel lymph or lymphadenectomy; RT: radiotherapy; S: surgery; CT: chemotherapy; NA CT: neoadjuvant chemotherapy; H: hormone therapy; M: male; ECT: electrochemotherapy.

## CONCLUSION

Radiogenic angiosarcoma of the breast is a rare and late complication of breast irradiation. Alterations may be confused with other benign skin conditions such as telangiectasia. The prognosis in women is poor. We presented a case to highlight clinical aspects and alert to the diagnostic possibility in patients with a previous history of adjuvant radiation therapy for breast cancer treatment.
