# Supplementary material for: Radiation-induced angiosarcoma: case report
Source: Einstein (Sao Paulo). 2020 Nov 25;18:eRC5439. doi: 10.31744/einstein_journal/2020RC5439 (PMC7690932; doi:10.31744/einstein_journal/2020RC5439)
Supplement: Translation

# Translation: Angiossarcoma induzido por radiação: relato de caso

**Authors:** Lucas Aguiar Alencar de Oliveira, Antonio Fortes de Pádua, Maria Adélia Medeiros e Melo, Elisa Rosa de Carvalho Gonçalves Nunes Galvão, Mharcus Carneiro Vieira, Jerúsia Oliveira Ibiapina, Danilo Rafael da Silva Fontinele, Sabas Carlos Vieira

**Affiliations:** 1 TeresinaPI Brasil Universidade Federal do Piauí, Teresina, PI, Brasil.; 2 TeresinaPI Brasil Hospital São Marcos, Teresina, PI, Brasil.; 3 TeresinaPI Brasil Universidade Estadual do Piauí, Teresina, PI, Brasil.

**Keywords:** Hemangiossarcoma, Neoplasias da mama/radioterapia, Mastectomia

## Abstract

Os angiossarcomas de mama representam menos de 1% dos tumores da mama e podem ser primários ou secundários à radioterapia prévia. Tais tumores são chamados de “angiossarcomas radiogênicos da mama” e representam uma entidade mais rara ainda e de prognóstico ruim. Atualmente, na literatura, são encontrados apenas 307 casos desses tumores. Relatamos o caso de uma mulher de 73 anos, com história prévia de tratamento conservador de câncer de mama direita, apresentando alteração rósea discreta em pele da mama homolateral. A mamografia demostrou resultado compatível com alterações benignas (BI-RADS 2). No material de biópsia incisional, a hematoxilina-eosina demonstrou lesão vascular atípica e sugeriu imuno-histoquímica para elucidação diagnóstica. A paciente foi submetida à ressecção de lesões, e a histologia demonstrou angiossarcoma radiogênico. Em seguida, mastectomia simples foi realizada. A imuno-histoquímica demonstrou positividade para os antígenos relacionados a CD31 e CD34, e a amplificação do oncogene C-MYC confirmou o diagnóstico de angiossarcoma induzido por radiação mamária. O atraso no diagnóstico constitui questão importante. Considerando que as alterações iniciais da pele do angiossarcoma radiogênico são sutis e podem ser confundidas com outras condições benignas da pele, como telangiectasias, neste relato destacamos os aspectos clínicos, no intuito de alertar sobre a possibilidade de angiossarcoma de mama em pacientes com história prévia de radioterapia adjuvante para tratamento de câncer de mama. A paciente permaneceu assintomática 16 meses após a cirurgia.

## INTRODUÇÃO

O angiossarcoma de mama representa menos de 1% dos tumores mamários. Tal tumor pode ser primário ou secundário à radioterapia anterior e/ou linfedema crônico após tratamento para câncer mamário.^(^^1^^)^

O angiossarcoma secundário de mama é geralmente relacionado à radioterapia contra o câncer de mama, sendo conhecido como angiossarcoma de mama radiogênico (AMRO). O risco de desenvolver AMRO é baixo (menor do que 0,5%), e o risco relativo de desenvolver essa condição é de 15,9 em pacientes submetidos à radioterapia para câncer de mama.^(^^2^^)^

As alterações precoces no AMRO são sutis e podem ser confundidas com outras condições benignas da pele, como a telangiectasias, que podem não chamar a atenção do médico durante o diagnóstico.

Apresenta-se um caso de AMRO e discutimos os aspectos do diagnóstico e do tratamento.

## RELATO DE CASO

Paciente do sexo feminino, caucasiana, 73 anos de idade que procurou um serviço de distúrbios mamários com queixa de alteração rósea na pele da mama direita há cerca de 30 dias. A mamografia bilateral 5 meses antes da consulta mostrou alterações benignas ( *Breast Imaging Reporting and Data System* – BI-RADS™ 2). A paciente apresentou histórico de carcinoma ductal invasivo na mama direita G3 sem tipo especial, que fora tratado com ressecção segmental e dissecção de linfonodos axilares níveis I e II em dezembro de 2011. No momento da cirurgia, observaram-se tumor medindo 3,0cm e três linfonodos axilares apresentando metástase (3/10). A imuno-histoquímica relevou que o tumor era positivo para receptor de estrógeno (90%), positivo para receptor de progesterona (2%), HER-2 negativo, Ki-67 positivo (60%) e do subtipo luminal B. A terapia adjuvante da paciente incluiu seis ciclos de doxorrubicina, ciclofosfamida e terapia com paclitaxel, seguida de radioterapia (25 sessões com dose de 50Gy na mama direita e fossa supraclavicular, além de *boost* de 10Gy). Atualmente, a paciente está em tratamento endocrinológico com letrozole (6 anos de tratamento). Em exame físico, ela apresentou duas lesões róseas na mama direita, sendo uma eritematosa leve e quase imperceptível, na junção do quadrante superior (JQS), e a outra uma lesão violácea mais intensa, na junção do quadrante inferior (JQI) medindo 0,5cm ( Figura 1 ).

**Figura 1:** Lesões róseas e violáceas, sendo uma localizada na junção do quadrante superior (A) e outra na junção do quadrante inferior, medindo 0,5cm (B)

Realizou-se biópsia incisional da lesão no JQI, que demostrou lesão vascular atípica em coloração de hematoxilina-eosina. Um estudo imuno-histoquímico complementar do angiossarcoma foi sugerido para conclusão do diagnóstico. Após o resultado desse estudo, a paciente foi submetida à ressecção de ambas as lesões cutâneas. O relato patológico anatômico do espécime ressecado mostrou angiossarcoma bem diferenciado (G1), tratando-se de neoplasia caracterizada por anastomoses vasculares alinhadas por células endoteliais atípicas caracterizada pela presença de hipercromatismo e anisocariose e, em alguns casos, contendo eritrócitos, organizados no padrão de crescimento infiltrado, permeando o parênquima mamário e a derme ( Figura 2 ) na JQS, medindo 1,9x1,4cm, e na JQI, constituindo neoplasia restrita à derme, medindo 1,1x0,5cm ( Figura 3 ). O exame imuno-histoquímico relevou positividade para expressão do grupamento de diferenciação 31 (CD31) e oncogene C-MYC, confirmando a malignidade secundária à terapia por radiação ( Figura 4 ). A tomografia computadorizada do tórax e abdômen e a cintilografia óssea não mostraram sinais de metástase distante. O paciente realizou mastectomia simples do lado direito, sem reconstrução como tratamento complementar. O relato final histopatológico mostrou angiossarcoma bem diferenciado (G1) e mais três focos microscópicos foram encontrados no parênquima mamário, sendo que o maior deles mediu 2mm. Dois anos após a cirurgia, a paciente não desenvolveu recidiva.

**Figura 2:** Anatomia patológica

**Figura 3:** Espécimes obtidas da lesão da pele ressecada na junção do quadrante superior e junção de quadrante inferior da mama direita

**Figura 4:** Estudo imuno-histoquímico mostrando resultado positivo para (A) grupo de diferenciação 31 e (B) c-Myc oncogene

## DISCUSSÃO

Ao contrário do angiossarcoma de mama, que afeta mulheres jovens, a média de idade das pacientes no diagnósticos de AMRO foi de 70 anos, e a média do período de latência entre a terapia radiação e o diagnóstico foi de 6 anos.^(^^3^^)^A patogênese do AMRO ainda não está clara.^(^^4^^)^

As duas maiores série de casos do AMRO, uma com 79 casos e a outra com 95, relataram que os 5 anos de sobrevida livre da doença (SLD) foram de 47% e de 62,6%, respectivamente.^(^^5^^,^^6^^)^

Porém, o AMRO apresenta alta taxa local de recidiva e metástase, sendo mais comum nos pulmões e no fígado, ocorrendo simultaneamente ou logo após as recorrências locais.^(^^7^^)^

As manifestações clínicas da AMRO são frequentemente descritas como lesões da pele pequenas, violáceas e similares à telangiectasia. Além disso, nódulos, placas e lesões também podem aparecer.^(^^3^^)^

A imagem do angiossarcoma não é específica. A mamografia e a ultrassonografia não apresentam características patognomônica. A ressonância magnética por imagem é considerada o exame de imagem mais promissor para os padrões de malignidade.^(^^8^^)^

O diagnóstico do angiossarcoma é feito por biópsia. As características histológicas do angiossarcoma primário da mama AMRO são indistinguíveis, exceto pelo envolvimento comum cutâneo no AMRO, assim como a alta proporção de tumores epiteliais menos diferenciados.^(^^8^^,^^9^^)^Até hoje, os três principais graus histopatológicos têm sido descritos pelo angiossarcoma: grau leve ou tipo 1 (G1), grau intermediário ou tipo 2 (G2) e alto grau ou tipo 3 (G3).^(^^1^^)^D'Angelo et al.,^(^^6^^)^demostraram que o grau do tumor parece não ter valor prognóstico, e mesmo as lesões de baixo grau podem metastizar. Espat et al.,^(^^10^^)^consideram que todos os angiossarcomas associados com radiação são considerados tumores de alto grau. Portanto, o grau do tumor não tem valor prognóstico no angiossarcoma de mama.

Em exame imuno-histoquímico, o angissarcomas são positivos para antígenos relacionados ao CD31, ao CD34 e a algumas podoplaninas para o diagnóstico de tumores pouco diferenciados.^(^^6^^)^Laé et al.,^(^^11^^)^encontraram uma amplificação de 5 a 20 do oncogene C-MYC em todos os angiossarcomas induzidos por irradiação de mama. Esses dados podem disponibilizar uma base para terapia adicional guiada.^(^^11^^)^A expressão e a amplificação do c-Myc em AMRO são também importantes no diagnóstico diferencial de lesões benignas chamadas de vasculares atípicas. A positividade dos antígenos relacionadas com CD31 e CD34 confirma o diagnóstico de angiossarcoma, além da expressão e da amplificação do c-MYC observadas no AMRO.^(^^3^^)^

A partir da perspectiva molecular, assume-se que mutações pontuais na BRCA-2 são causas de alguns angiossarcomas secundários da mama. A perda da função do BRCA mutado evita que se exerça proteção contra a lesão no DNA induzida por radiação.^(^^12^^)^West et al.,^(^^13^^)^apresentaram relato de caso em que um paciente com BRCA-2 desenvolveu angiossarcoma na parede do toráx após mastectomia. Posteriormente, Kadouri et al.,^(^^14^^)^relataram avaliação genética de três casos de angiossarcoma secundário da mama, dois BRCA-1, um BRCA-2 e um caso sem a doença. Estima-se aproximadamente o dobro de risco do angissarcoma em pacientes com BRCA-1/2. Porém, esse risco não pode ser considerado na radioterapia da população com a mutação.^(^^12^^)^

Não há padrão-ouro para tratamento cirúrgico do angiossarcoma. Uma ressecção local extensa é o tratamento mais comumente realizado. A mastectomia simples é o tratamento cirúrgico de escolha. Discute-se a necessidade da dissecção axilar, considerando que o envolvimento nodal é incomum.^(^^5^^)^

Em geral, o regime quimioterápico é escolhido empiricamente, devido à raridade da doença e à falha do tratamento padronizado definitivo. Alguns estudos^(^^5^^,^^9^^)^têm sugerido que o tratamento com quimioterapia baseada em antraciclina, tanto com doxorubicina, quanto com epirrubicina e ifosfamida, pode melhorar tanto a SLD livre de doença quanto a sobrevida global (SG). A quimioterapia sistêmica e a reirradiação são indicadas somente em pacientes com AMRO com recidiva local e/ou sistemática.^(^^3^^)^

A tabela 1 apresenta relatos e séries de casos (até dez casos) dos últimos 5 anos (2015-2020) recuperados no PubMed.gov e relacionados ao angiossarcoma de mama induzido após tratamento para câncer de mama.

**Tabela 1:** Angiossarcoma de mama induzido por radiação após tratamento para câncer de mama^(^^15^^-^^39^^)^

Autor	Sexo/idade (ano)	Número de casos	Tratamento de tumor primário	Tamanho do angiossarcoma/tempo de radioterapia anterior	Seguimento (meses)	Resultado	Tratamento para angiossarcoma	Recidiva/tratamento
Abbenante et al.^(^^15^^)^	F/70	1	CB + L + RT	14 anos	4	Sem doença	C	
Shiraki et al.^(^^16^^)^	F/72, F/80	2	C + RT/C	18cm/5 anos, 3 anos	32/17	Mortes/ resposta parcial	C/C	Sim/QT
Jayarajah et al.^(^^17^^)^	F/62	1	CB + L + RT	0,5cm/5 anos	15	Sem doença	CB + QT	
Lewcun et al.^(^^18^^)^	F/64	1	CB + QT + RT	0,8cm/6 anos	24	Resposta completa	QT NA + CB	
Kong et al.^(^^19^^)^	F/75	1	S + L + QT+ RT + H	5,6cm/20 anos	15	Sem doença	C + RT	
Suzuki Y et al.^(^^20^^)^	F/62	1	C + RT+ H	8 anos	8	Não recorrência	C + QT	Sim
Amajoud et al.^(^^21^^)^	F/73 *	10	C + RT	10cm/7,3 anos *	13 *	5 mortes e 5 sem a doença	C + QT + RT	Sim
Lee et al.^(^^22^^)^	F/72	1	C + RT	6 anos				
Verdura et al.^(^^23^^)^	F/79	1	C + L + QT + RT	2cm/8 anos	12	Sem doença	QT NA + S	
Tsapralis et al.^(^^24^^)^	M/72	1	C + L + RT	6 anos		Morte	C + EQT + QT	Sim
Wei et al.^(^^25^^)^	F/39	1	C + RT	4 anos			C	
García Novoa et al.^(^^26^^)^	F/37	1	CB + L + QT + H + RT	0,5cm/4 anos			C	
Bonzano et al.^(^^27^^)^	F/57	1	C + L + RT	10cm/8 anos	30	Sem doença	C + QT + RT	
Farran et al.^(^^28^^)^	F/67	1	C + L + RT	1cm/8 anos		Sem doença, em seguimento	C	
Disharoon et al.^(^^29^^)^	F/68	1	C + L + RT	1cm/9 anos			C	
Plichta et al.^(^^30^^)^	F/72	1	C + RT	10cm/5 anos	12	Sem doença	C + QT	
Tato-Varela et al.^(^^31^^)^	F/62	1	C + L + RT	1cm/8 anos	0,5	Assintomático	C	
Wronski et al.^(^^32^^)^	F/56	1	C + L + RT	5 anos	0,06	Assintomático	C	
Wilhelm et al.^(^^33^^)^	F/70 *	7	C + RT	8,5 anos *				
Mocerino et al.^(^^34^^)^	F/77	1	C + H + RT	2cm		Sem doença	C + EQT + RT + QT	Sim/ RT + QT
Peterson et al.^(^^35^^)^	F/72	1	C + RT	1,5cm/14 anos	20	Sem doença	C	
Tidwell et al.^(^^36^^)^	F/68	1	RT	3cm/9 anos			C	Sim
Uryvaev et al.^(^^37^^)^	F/78 *	6	C + RT	9,2 anos	41,8	4 sem a doença, 1 morte, 1 at QT	C + QT + 3 RT	Sim (3)
Parvez et al.^(^^38^^)^	F/55	1	C + L + QT + RT + H	1,5cm/0,5 anos			CB + RT	
Styring et al.^(^^39^^)^	F/54,5 *	6	C + RT + 2 QT	7 anos *			C	

* Média.

F: sexo feminino; CB: cirurgia bilateral; L: linfonodo sentinela ou linfadenectomia; RT: radioterapia; C: cirurgia; QT: quimioterapia; QT NA: quimioterapia neoadjuvante; H: terapia hormonal; M: sexo masculino; EQT: eletroquimioterapia.

## CONCLUSÃO

O angiossarcoma radiogênico da mama é raro e uma complicação tardia da irradiação da mama. Alterações podem ser confundidas com outras condições benignas da pele, como a telangiectasia. O prognóstico para mulher é ruim. Apresentamos este caso com o objetivo de enfatizar aspectos clínicos e alertar sobre a possibilidade diagnóstica em pacientes com histórico prévio de radioterapia adjuvante para tratamento de câncer de mama.
